# The role of mechanically sensitive ion channel Piezo1 in bone remodeling

**DOI:** 10.3389/fbioe.2024.1342149

**Published:** 2024-02-08

**Authors:** Yugui Du, Bowen Xu, Quiying Li, Chuhan Peng, Kai Yang

**Affiliations:** Department of Orthodontics, School of Stomatology, Capital Medical University, Beijing, China

**Keywords:** Piezo1, bone remodeling, osteoblasts, osteoclasts, mechanical force

## Abstract

Piezo1 (2010) was identified as a mechanically activated cation channel capable of sensing various physical forces, such as tension, osmotic pressure, and shear force. Piezo1 mediates mechanosensory transduction in different organs and tissues, including its role in maintaining bone homeostasis. This review aimed to summarize the function and possible mechanism of Piezo1 in the mechanical receptor cells in bone tissue. We found that it is a potential therapeutic target for the treatment of bone diseases.

## 1 Introduction

Mechanotransduction, a fundamental process conserved throughout evolution, refers to the ability to sense mechanical force and convert it into biochemical signals, ultimately achieving complex physiological functions, such as blood pressure regulation and lung relaxation. The discovery of Piezo channels in 2010 improved the understanding of the molecular and cellular mechanisms of mechanotransduction (Coste et al., 2010). The Piezo family, comprising Piezo1 and Piezo2, has the unique ability to rapidly convert diverse mechanical inputs, including tension, osmotic pressure, and shear stress, into electrical impulses.

Piezo1 is a mechanosensitive ion channel that plays a crucial role in bone remodeling, a process that involves the removal of old or damaged bone by osteoclasts and subsequent replacement with new bone formed by osteoblasts. Piezo1 is found in tissues throughout the body, including bone, and is involved in sensing changes in mechanical stress (Sun et al., 2019). Notably, Piezo1 is closely related to the development of osteoporosis (OP) (Xu et al., 2021). Furthermore, Piezo1 is expressed in both condylar cartilage and subchondral bone (Wu et al., 2022). The inhibitor GsMTx4 has recently attracted much attention as a promising treatment for cartilage injury (Li et al., 2016). GsMTx4 can weaken Piezo-mediated mechanically activated (MA) currents and reduce chondrocyte death induced by mechanical force (Lee et al., 2014).

Piezo1 is expressed and functions in various mechanical sensor cells, including osteoblasts (Sun et al., 2019; Song et al., 2020), osteoclasts (Wang et al., 2020), osteocytes (Liu Z. et al., 2022), bone marrow mesenchymal stem/stromal cells (BMSCs) (Zhou et al., 2020), chondrocytes (Hendrickx et al., 2021), periodontal ligament fibroblasts (PDLFs) (Kang et al., 2014), and periodontal ligament stem cells (PDLSCs) (Lin Y. Y. et al., 2022). These cells participate in bone formation and resorption, ultimately maintaining bone homeostasis. This study presents an overview of the structure and properties of Piezo channels, mainly focusing on recent advancements in understanding the role of Piezo1 in bone remodeling. Additionally, we explored potential signaling pathways associated with Piezo1.

## 2 Piezo1

### 2.1 Discovery of the Piezo family

Previous studies have demonstrated that MA cation channels, considered a specialized subset of mechanotransducers, are ubiquitously expressed in various cell types and can be triggered by various mechanical forces. These channels can promptly initiate cellular responses after activation. Although TRP ion channels and DEG/ENaC channels significantly promote invertebrate mechanotransduction, the mechanisms underlying mechanotransduction in mammals are unclear (Ranade et al., 2015). Therefore, identifying MA cation channels in mammals is crucial for enhancing the understanding of the mechanotransduction mechanism.

In 2010, Patapoutian and colleagues made a groundbreaking discovery: the Piezo ion channel family, comprising Piezo1 and Piezo2 (Coste et al., 2010). In that study, a significant increase in mechanosensitive currents was detected in a specific mouse neuroblastoma cell line known as Neuro2A cells. They found that the Piezo1 gene, also known as Fam38A, is essential for generating these MA currents based on RNA interference techniques. Furthermore, they found that Fam38B can encode the Piezo2 protein through homologous sequence analysis.

### 2.2 Structure of Piezo1

**FIGURE 1:**
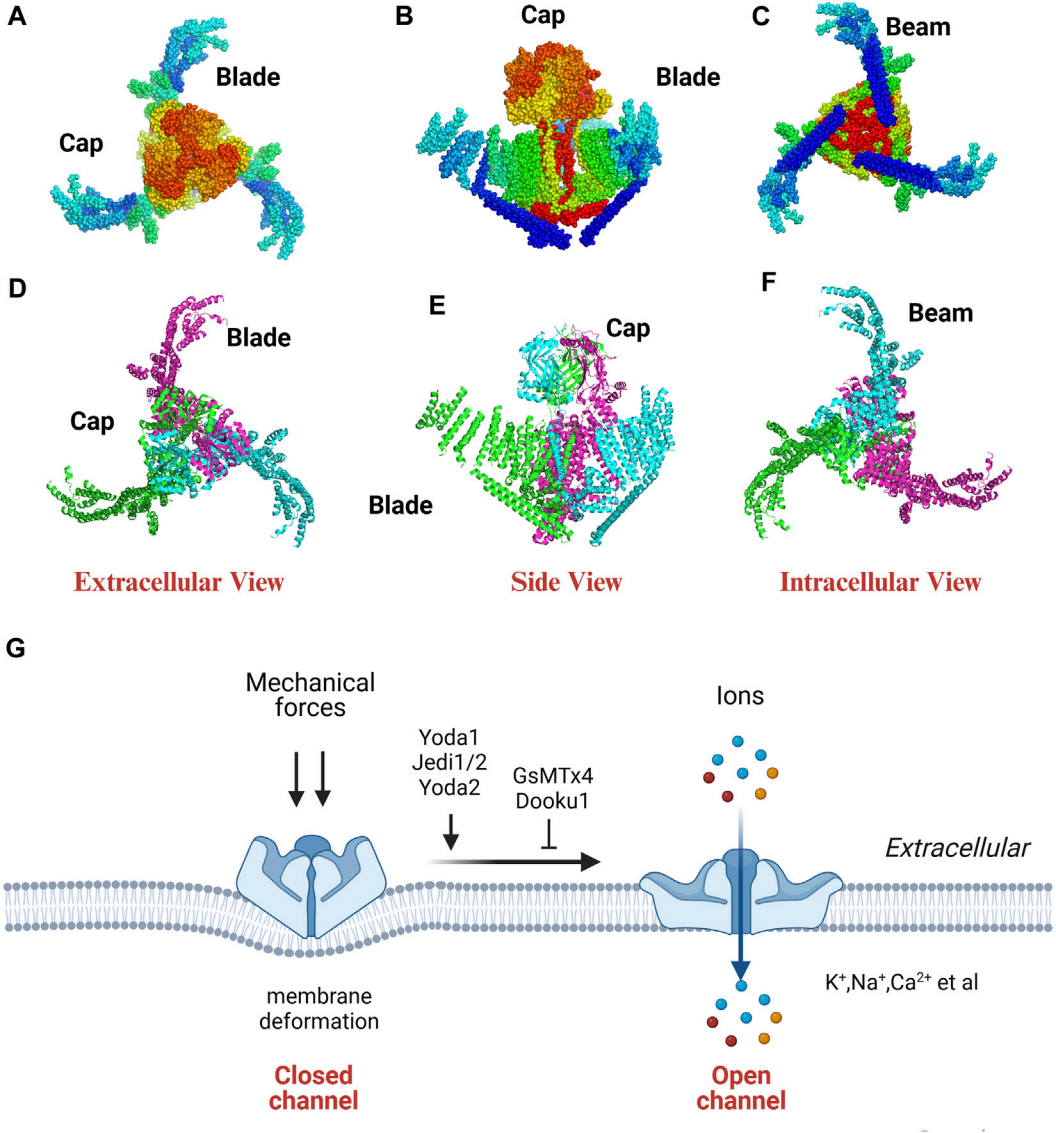
The cryo-EM structure **(A–F)** and property **(G)** of Piezo1. The extracellular, side, intracellular view of the Piezo1 channel indicated by dots **(A–C)** and cartoon model **(D–F)**. PDB ID: 3jac; cited from Ge et al. (2015).

Ge et al. (2015) unveiled the high-resolution three-dimensional configuration of mouse Piezo1 using cryo-electron microscopy (cryo-EM), which resembles a three-bladed propeller (Figure 1). Piezo proteins consist of a central cap and three distal blades on the extracellular side and three elongated beams (length; about 90 nm) on the intracellular side. The transmembrane (TM) region is situated between the domains exposed on the extracellular and intracellular surfaces. The beams connect peripheral TMs and blades, linking them to the lower central axis of the channel complex. The TM region exhibits significant curvature and clockwise twist, akin to the wing-shaped blade found in propellers, comprising 9 TM helical units (THUs) (Ge et al., 2015; Zhao et al., 2016; Zhao et al., 2018).

Piezo channels are dissected into two distinct functional modules (a central ion-conducting pore and a mechanotransduction module) to elucidate the correlation between the structure and function of the Piezo1 channel. The ion-conducting pore module has three primary components: the C-terminal extracellular domains (CEDs), the TM inner helices (IHs) and outer helices (OHs), and the intracellular C-terminal domains (CTDs). This module regulates ion selectivity, unitary conductance, and pore obstruction. The mechanotransduction module comprises the extracellular distal blades, the peripheral helices (PHs), the TM anchors, and the intracellular beams (Wang and Xiao, 2018). This module plays a role in the detection have a record-breaking 114 TMs based on a novel 38-TM topology (Xiao, 2020). The three large TM blades act as mechanosensors, detecting alterations in membrane tension and influencing the conformational arrangement of the channel.

Zhao et al. (2018) introduced a mechanogating mechanism that resembles a lever, categorizing the intricate and efficient process of long-distance mechanotransduction. Residues L1342 and L1345 function as pivotal points of the lever, positioned at a greater distance from the TM blade at the distal end while being closer to the central pore module of Piezo1 at the opposite end. Based on the lever principle, applying a lighter force to the TM blade with a longer force arm amplifies output force, facilitating gating of the central ion-conducting pore and selective cation penetration (Wang Y. et al., 2018; Zhao et al., 2019; Xiao, 2020). The assembled propeller-shaped machine effectively converts significant conformational alterations of the distal blades into a subtle movement of the core pore structure, transforming mechanical stimulation into ionic influx.

However, further studies should assess the mechanisms by which mechanical force modulates the activity of Piezo channels. The structure-based membrane dome mechanism suggested that Piezo protein deforms the membrane locally into a dome shape when it is in a closed state. However, this dome undergoes a relative flattening upon the application of a force. The transition of Piezo channels from a closed to an open state enhances its ability to respond to mechanical stimuli (Guo and MacKinnon, 2017). Geng et al. (2020) introduced a “plug-and-latch” mechanism in which Piezo1 outfitted three lateral ion-conducting portals with three independently positioned lateral plug gates which were strategically secured to the central axis to achieve synchronized gating of the three portals. The coordinated action of plugs and latches in the Piezo1 channel is impacted by mechanical forces acting asymmetrically on the force-sensing blades in this mechanism. Wang J. et al. (2022) found that the Piezo channels have a biochemical and functional connection to the actin cytoskeleton through the cadherin-β-catenin mechanotransduction complex. As a result, Piezo channels can effectively detect and respond to long-range mechanical disturbances within a cell.

Yang et al. (2022) elucidated the structural characteristics of Piezo1 in both curved and flattened conformations within liposome vesicles using cryo-EM. They found that Piezo1 protein has a curved conformation that can flatten. Furthermore, the protein beam can bend under mechanical stimulation while the protein cap can detach and rotate in response to mechanical stimulation. These alterations can facilitate the opening of the ion-conducting pathway, thus regulating the channel. The remarkable mechanosensitivity and specific curvature-based gating observed in lipid membranes could be due to the deformability and structural rearrangement of Piezo1. Mulhall et al. (2023) directly visualized and quantified the conformational dynamics of individual Piezo1 molecules within a cellular context using nanoscopic fluorescence imaging techniques. They found that Piezo1 blades can significantly expand while in a resting state due to the bending tension applied by the plasma membrane. Besides, the blades exhibited varying degrees of rigidity along the length. Stiffness increased at the base while the flexibility increased towards the ends. The researchers also investigated the correlation between blade growth and the activation and inhibition of channels. They found that the inhibitor of Piezo1, or the removal of the plasma membrane, can increase compaction levels in the blades. Moreover, Piezo1 activation, either through osmotic or chemical stimulation slightly expanded the blades (1–2 nm increase). These findings reveal that the conformation of the Piezo1 is more intricate than initially anticipated based solely on modeling.

### 2.3 Piezo1 regulation

Piezo1 protein can sense various forces, such as tension, poke force, osmotic pressure, and fluid shear force and convert mechanical stimuli into electrical signals in milliseconds (Retailleau et al., 2015). Piezo1 is a non-selective cation channel permeable to K^+^, Na^+^, Ca^2+^, and Mg^2+^, with a slight preference for Ca^2+^ (Coste et al., 2010) (Figure 1).

Piezo1 channels can perceive various mechanical stimuli in distinct manners. Atomic force microscopy (AFM) experiments have revealed that Piezo1 channels have distinct responses to pushing and pulling forces (Gaub and Müller, 2017). Specifically, pulling forces can more efficiently activate Piezo1 channels in the presence of extracellular matrix (ECM) proteins. Ozkan et al. (2023) monitored the local rearrangements occurring along the blades of Piezo1 under varying forces by inserting two cyclic permuted green fluorescent proteins as a probe. Significant fluorescence signals were observed from the probes upon Piezo1 activation by low-intensity fluid shear stress (FSS). However, no visible fluorescence signals were produced by the Piezo1 channel activation in response to cellular indentations, osmotic swelling, and high-intensity flow stimuli.

Piezo1 channels can be strongly regulated by voltage in addition to mechanical stimuli, and can even transition to a solely voltage-gated mode (Moroni et al., 2018). Moreover, stomatin-like protein 3 (STOML3) can significantly regulate Piezo channels by reducing the activation threshold of Piezo1 and Piezo2 currents (Poole et al., 2014).

Yoda1, Jedi1/2 and Yoda2 can activate Piezo1 channel. Jedi1/2, as a synthetic agonist, can activate Piezo1 by binding to the upstream blade (Wang Y. et al., 2018). Yoda1, as a synthetic small molecule, activates Piezo1 by acting as a molecular wedge, inserting itself between 2 TM regions (Syeda et al., 2015; Botello-Smith et al., 2019; Jiang et al., 2023). Yoda2, 4-Benzoic acid modification of Yoda1, can more effectively activate Piezo1 channel with better solubility and stability (Parsonage et al., 2023).

Ruthenium red, gadolinium, streptomycin, and GsMTx4 (grammostola spatulata mechanotoxin 4) can inhibit Piezo channels. Ruthenium red, gadolinium, and streptomycin are nonspecific inhibitors of Piezo1, blocking multiple cationic channels. GsMTx4, a peptide derived from spider venom, can selectively block Piezo and TRP channel families by modulating lipid bilayer fluidity within the membrane (Bae et al., 2011). Dooku1, a Yoda1 analogue, acts at the same location as Yoda1 to efficiently inhibit Yoda1-induced Piezo1 channel activation. However, Dooku1 cannot inhibit constitutive Piezo1 channel activity (Evans et al., 2018). Similarly, Tubeimoside I (TBMS1), a compound obtained from Traditional Chinese Medicine, can inhibit Yoda1-induced activation of Piezo1 channels (Liu et al., 2020).

### 2.4 The function of Piezo1

**FIGURE 2:**
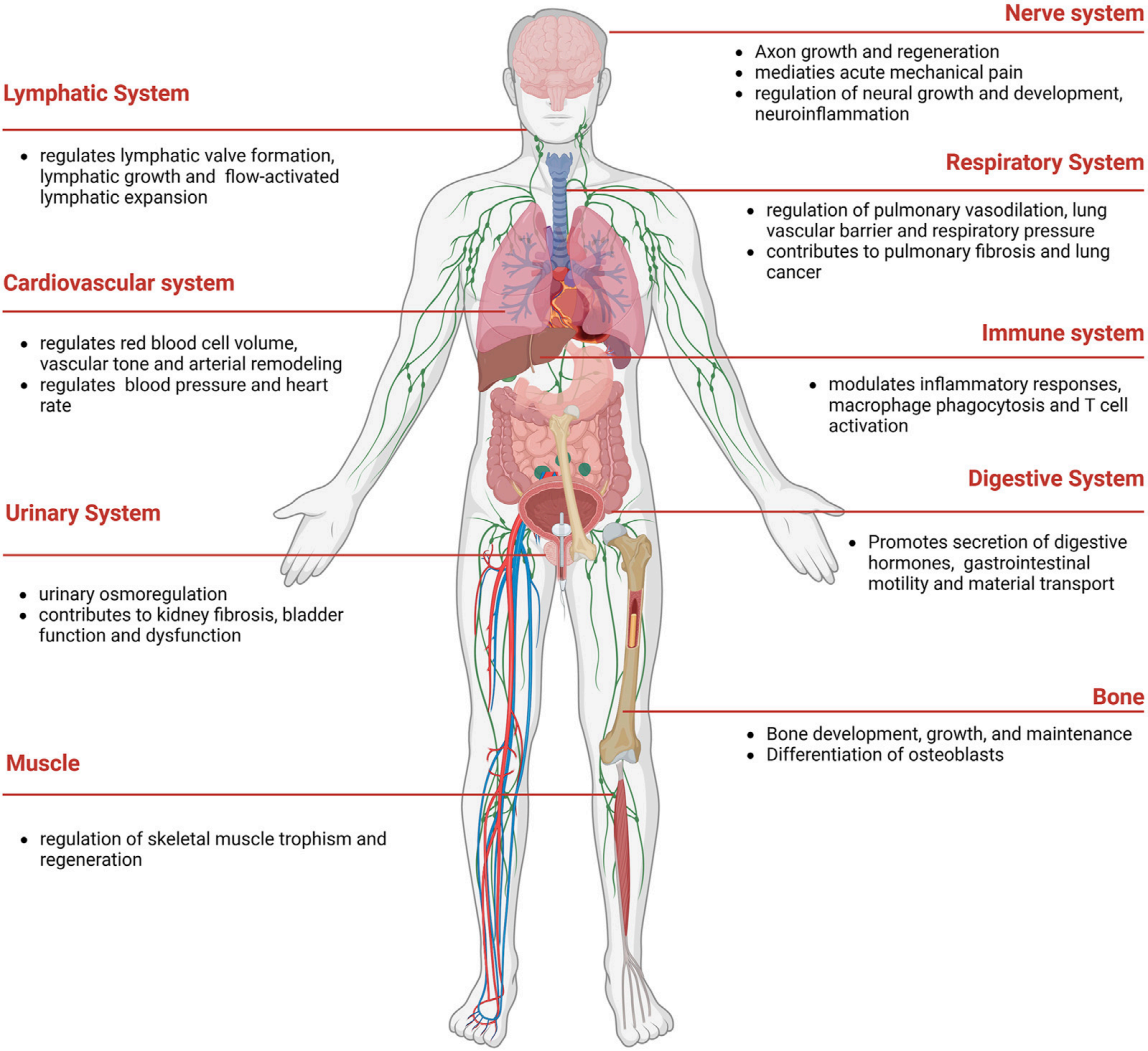
The distribution of Piezo1 in human.

Piezo proteins, comprising approximately 2,500–2,800 amino acids, exhibit remarkable evolutionary conservation and lack substantial sequence homology with known ion channels (Coste et al., 2010). Notably, Piezos can detect various mechanical stimuli and produce rapid cationic currents in different mammalian cell lines (Table 1). Piezo1 is abundantly present in various organs, such as lung, bladder, intestines, and skeleton tissue. Besides, Piezo1 participates in cardiovascular mechanical transduction, immune regulation, epithelial cell homeostasis, red blood cell volume regulation, and bone formation (Coste et al., 2010) (Figure 2). Piezo2 is significantly expressed in sensory tissues, including dorsal root, trigeminal ganglia sensory neurons, and Merkel cells, where they primarily respond to touch and proprioception (Geng et al., 2016; Gottlieb, 2017).

**TABLE 1:** Piezo1 distribution and function.

Tissue	Cell	Mechanical stimulation	Function	Reference
Vascular system	Endothelium and smooth muscle cells, blood cell	FSS	Vascular development; blood pressure regulation; red blood cell volume regulation	Ranade et al. (2014), Retailleau et al. (2015), Wang et al. (2016), Gudipaty et al. (2017), Murthy et al. (2017), Wong et al. (2018), Beech and Kalli (2019)
Lymphatic system	Lymphatic endothelial cells	FSS	The development and maintenance of lymphatic valves	Nonomura et al. (2018), Choi et al. (2019)
Lung	Alveolar capillary endothelial cells	Alveolar pressure and hydrostatic pressure (HP)	Maintain lung function	Mammoto et al. (2022), Grannemann et al. (2023)
Nerve system	Retinal ganglion cells, neural stem cells	Stretch	Axon growth and regeneration, directs the differentiation of neural stem cells	Koser et al. (2016), Wu et al. (2017)
Gastric mucosa	G cells	Antrum distension	Regulate gastrin secretion	Lang et al. (2018)
Intestines	Intestinal epithelial	HP and shear force	Regulate epithelial function and permeability	Jiang Y. D. et al. (2021), He et al. (2023)
Bladder, and kidney	Bladder and kidney epithelial cells	Shear stress and wall tension	Sense bladder distension and urinary osmolarity, concentrate urine	Michishita et al. (2016), Dalghi et al. (2019)
Tooth	Odontoblasts, dental pulp stem cells (DPSC), Oral squamous cell (OSC)	Intrapulpal pressure changes, extracellular matrix stiffness	Regulate DPSC and OSC proliferation, pulpitis attack and dentin mineralization	Gao et al. 2017, Sato et al. (2018), Hasegawa et al. (2021), Matsunaga et al. (2021)
Cartilage	Chondrocytes	Osmotic stress	Cartilage mechanotransduction	Lee et al. (2014)

Piezo1 mutations are linked to certain hereditary human diseases, including dehydrated hereditary stomatocytosis (DHS) (Andolfo et al., 2013) and generalised lymphatic dysplasia (GLD) (Lukacs et al., 2015). GLD is caused by loss-of-function mutations in the Piezo1 gene (Martin-Almedina et al., 2018), characterized by various symptoms, including non-immune hydrops fetalis (NIHF), lymphedema, and recurrent cellulitis (Chen Y. et al., 2021). Loss-of-function mutations in the Piezo1 gene, especially S217L and G2029R, have been shown to alter protein stability due to increased ubiquitination and subsequent proteasomal degradation (Zhou et al., 2021). Furthermore, Piezo1 loss-of-function compound heterozygous mutations have been reported in patients with Prune Belly Syndrome, which is characterized by a “Prune-like” wrinkled, flaccid ventral abdominal wall with regionally missing or hypoplastic skeletal muscle (Amado et al., 2024).

Meanwhile, DHS is associated with gain-of-function mutations in the Piezo1 gene (Fotiou et al., 2015). A recent case report showed that the c.7505A>G variant can cause a DHS phenotype. This variant is cataloged in the single nucleotide polymorphism database (dbSNP) with the reference ID rs34830861 (Martin-Almedina et al., 2018). Another gain-of-function mutation in Piezo1 gene, known as E756del has been linked to enhanced athletic performance and the ability of protection against severe malaria. When performing leaping actions requiring high tendon loading, energy storage, and return, carriers of the E756del mutation outperform non-carriers by a large margin (Passini et al., 2021).

Recent case reports have highlighted skeletal manifestations associated with Piezo1 mutations, demonstrating the diverse impact of this gene on human health. Lee et al. (2021) reported a case of a 21-year-old male diagnosed with primary lymphatic dysplasia who exhibited additional clinical features, such as a history of multiple fractures in infancy, thoracolumbar scoliosis, low height, and hypoplasia of the left-sided facial bones. Exome analysis revealed that the patient had two previously unreported pathogenic variants of Piezo1 in a trans configuration, including a heterozygous deletion spanning 93.7 kb (chr16:88,782,477-88,876,207; exon 1-50) and a single nucleotide substitution c.2858G>A (p.Arg953His). This case of compound heterozygosity, where the variants were inherited from different parents, underscores the potential impact of Piezo1 mutations on bone health. Another Piezo1 gene mutation was identified in the 61-year-old male patient with non-transfusion secondary hemochromatosis, specifically the missense mutation c.C4748T (p.A1583V). Osteoarticular involvement was indicated by thinning bone cortices and an enlarged tibial medullary cavity by computed tomography. However, it’s important to note that the search results do not provide direct evidence that the patient’s osteoarticular phenotype is caused by this mutation, or iron deposition due to the iron overload condition. To fully comprehend the possible involvement of the Piezo1 gene in bone health and illness, more investigation is required (Ruan et al., 2020).

## 3 Piezo1 in bone cells

**FIGURE 3:**
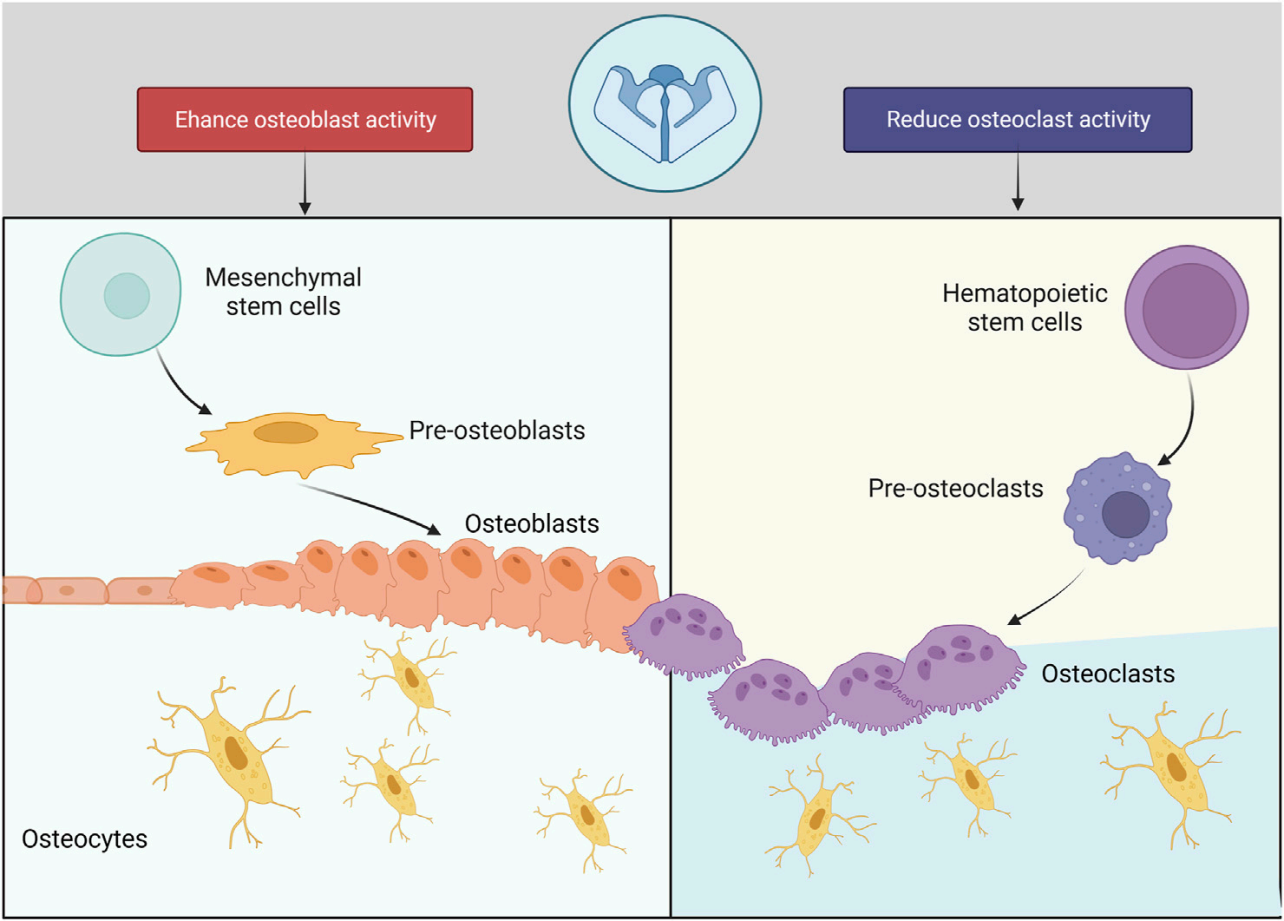
Piezo1 in bone cells.

Piezo1 regulates skeleton homeostasis in osteoblast lineage cells (Figure 3). The elimination of Piezo1 in mice results in fatal outcomes, due to disruption of vascular development (Ranade et al., 2015). Conditional Piezo1 deletion by various Cre strains in Osteoblast lineage cells showed reduced trabecular and cortical bone mass (Nie and Chung, 2022; Dienes et al., 2023) (Table 2).

**TABLE 2:** Piezo1 function in bone cells.

Animal	Phenotype	Animal condition	Cell	Cell condition	Function	Signaling	Reference
Piezo1 Dmp1-Cre mice	Decreased cortical thickness, spontaneous tibial fracture		MLO--Y4, bone marrow macrophages	Yoda1	Piezo1 suppresses age--associated bone resorption	Through Ca^2+^/CaM/mTOR pathway	Li et al. (2023)
Piezo1 Lyz2-Cre; Dmp1-Cre; Col2a1-Cre; Runx2-Cre mice	Reduced trabecular and cortical bone mass; secondary spongiosa development abnormality; Aberrant osteoblast morphology		Primary osteoblasts, MC3T3-E1, ATDC5 cells	Shear stress, Yoda1	Piezo1 plays an essential role in endochondral ossification and bone remodeling		Hendrickx et al. (2021)
Piezo1 Prx1-Cre; Sp7-Cre mice	Multiple bone fractures, reduced trabecular and cortical bones		Primary Mouse BMSCs	FSS, Yoda1, matrix rigidity	Piezo1 is essential for bone development and osteoblast differentiation	Through NFAT-YAP1-ß-Catenin pathway	Zhou et al. (2020)
Piezo1 Dmp1- Cre mice	Reduced bone volume of the mandible and maxilla; loss of the vertical alveolar bone height; increased osteoclasts number; no significant differences in tooth movement distance				Piezo1 is crucial for osteoclast function		Nottmeier et al. (2023)
Male C57BL/6 wild-type mice	Poor bone; remodeling, fewer bone trabeculae	Exercise on the treadmill and GsMTx4 treatment	BMSCs, RAW264.7 cells	Cyclic tensile strain (CTS), Yoda1	Piezo1 promotes BMSCs proliferation, migration and osteogenic differentiation by induced M2 macrophage polarization	Through P53	Cai et al. (2023)
C57BL/6J mice	Rescued Bone Loss by Yoda1	Hindlimb unloading mouse model; OVX-induced osteoporosis and aging male mouse models	BMSCs	Yoda1	Piezo1 promotes the proliferation and osteogenic differentiation of BMSCs and related to bone loss especially under unloading	Piezo1/β-catenin/ATF4 Axis	Hu Y et al. (2023)
Medaka fish	Impaired caudal fin ray development	HP loading	UE7T-13, SDP11, Saos-2, HuO9, MG63, MC3T3-E1, Primary human MSCs	HP loading, Yoda1	Piezo1 regulates osteoblast differentiation and adipocyte differentiation of MSCs under HP pressure	Through BMP2	Sugimoto et al. (2017)
SD rats	Smaller damage to the cartilage and subchondral bone of the Piezo1 inhibitor group	Temporomandibular joint osteoarthritis animal model (TMJ-OA models)			Piezo1 regulates the condylar bone and subchondral bone destruction	Through pSmad3	Wu et al. (2022)
Piezo1flox/flox; AggrecanCreERT2 mice	Decreased meniscus ossification and osteophyte formation; significant reductions in cartilage erosion, proteoglycan loss, osteophyte and synovial formation and an increase in OARSI score in articular cartilage	Destabilization of medial meniscus (DMM)induced OA model	Human primary articular chondrocytes		Piezo1 inactivation slows the development and progression of OA.	Through PI3K-AKT	Gan et al. (2023)
			MC3T3-E1	FSS	Piezo1 regulates osteogenesis	Through AKT/GSK-3β/β-catenin pathway	Song et al. (2020)
			MC3T3-E1	Static magnetic field	Piezo1 promotes osteogenic differentiation		Hao et al. (2019)
			MC3T3-E1	Direct mechanical stimulation to cell membrane by the pipette, Yoda1	Piezo1 responds to mechanical stimulation		Nagai et al. (2023)
			MC3T3-E1	Low-intensity ultrasound stimulation (LIPUS)	Piezo1 promotes migration and proliferation ability	Activate ERK1/2 phosphorylation and perinuclear F-actin filament polymerization	Zhang et al. (2021)
			MLO-Y4 osteocytes	FSS, Yoda1	Piezo1 promotes OPG and inhibits RANKL	Notch 3	Liu Z et al. (2022)
			MLO-Y4 osteocytes	FSS, Yoda1	Piezo1 activates connexin 43 hemichannels in bone	Through PI3K signaling pathway	Zeng et al. (2022)
			IDG-SW3	Cyclic stretching, Yoda1	Piezo1 downregulates Sost expression	Piezo1-Akt pathway	Sasaki et al. (2020)
			DPSCs, PDLSCs	LIPUS	Piezo1 promotes cell proliferation	MAPK signaling	Gao et al. (2017)
			Human periodontal ligament fibroblasts	Compression force	Piezo1 is activated by compression force and then induces ATP release		Horie et al. (2023)
			DPSCs	Yoda1	Piezo1 regulates MSC migration	PYK2 and MEK/ERK signaling pathways	Mousawi et al. (2020)
			Human dental follicle cells	Yoda1	Piezo1 enhances the osteogenic differentiation	Wnt/β-catenin signaling pathway	Xing et al. (2022)

### 3.1 Piezo1 in BMSCs

The BMSCs can differentiate into osteogenic, adipogenic, and chondrogenic lineages under different loading conditions (Pierce et al., 2019). Osteoblast differentiation from BMSCs occurs through various intersecting signaling pathways, including Wnt pathways, bone morphogenetic protein (BMP) pathway (James, 2013), and specific transcription factors, including runt-related transcription factor 2 (Runx2) (Zhang et al., 2010; Sun et al., 2021a).

Piezo1, in particular, has been identified as a critical mechanotransducer in various biological processes, including bone formation. It is expressed in differentiating osteoblasts and hypertrophic chondrocytes in developing skeletal structures, and its expression increases during postnatal development following elevated mechanical stress (Zhou et al., 2020). The Piezo1 agonist Yoda1 promoted bone formation and osteoblast differentiation in developing mouse limb buds even under static conditions. In contrast, Piezo2 appears to have a more redundant and dispensable function during bone formation. The deletion of Piezo2 in osteoblasts had little adverse effect on skeletal development (Zhou et al., 2020).

The absence of Piezo1 in the mesenchyme of developing limbs achieved by the utilization of Prx1-Cre result in many skeletal abnormalities in mice, including shortened long bones, diminished quantities of trabecular and cortical bone, and increased risk of spontaneous bone fractures in both newborn and early adult mice (Wang et al., 2020; Zhou et al., 2020). The observed effects are associated with heightened osteoclast function and diminished osteoblast function, as evidenced by a decrease in procollagen type I N-terminal propeptide (P1NP) and Osterix levels. Interestingly, the low bone mass phenotype in Piezo1 Prx1-Cre mice appears to be limited to long, load-bearing bones. The calvariae are not affected by Piezo1 deletion, likely due to their lower load-bearing capacity compared to long bones. To elucidate the role of mechanical stimulus in bone formation, a mechanical unloading model through tail suspension was established, revealing a reduction in bone mass in the control group, but no such effect was observed in Piezo1 Prx1-Cre mice (Wang et al., 2020).

Piezo1 plays a crucial role in BMSCs differentiation under mechanical stimulation. Sugimoto et al. (2017) discovered that hydrostatic pressure (HP) enhances the expression of Piezo1 and BMP2 in human BMSCs, thus promoting osteoblast differentiation and inhibiting adipocyte differentiation. Yoda1 treatment can significantly alleviate bone loss caused by microgravity and aging and also promote the proliferation and osteogenic differentiation of BMSCs (Hu et al., 2023). The researchers designed a wearable pulsed triboelectric nanogenerator powered by human body motion, to activate Piezo1 channel and upregulate osteogenic differentiation potential of aging BMSCs. This finding provides a possible target for bone regeneration, especially for aged people (Wang B. et al., 2022). Moreover, static magnetic field (SMF) can enhance BMSC migratory capacity through Piezo1 (Sun et al., 2023). Piezo1 also regulates the differentiation ability of BMSCs into chondrocytes. Notably, exosomes produced by siRNA-Piezo1-treated BMSCs promote BMSC development into cartilage, thus enhancing the restoration of injured cartilage in osteoarthritis (OA) (Li et al., 2021).

### 3.2 Piezo1 in osteoblasts

Osteoblasts are primarily found in mesenchymal stem cells (MSCs) located within and outside the periosteum and within the bone marrow matrix (Abdallah et al., 2015).

Runx2 regulates the commitment of MSCs to the osteoblastic lineage during bone development. Mice lacking Piezo1 in Runx2-expressing cells (Piezo1 Runx2-Cre) exhibited several bone abnormalities including multiple spontaneous fractures, shorter femurs, pelvic dysplasia and a considerable decrease in trabecular bone mass below the growth plates. Similarly, Piezo1 Runx2-Cre mice exhibit no calvarial bone defects at birth nor changes in calvarial thickness (Hendrickx et al., 2021). Osterix (also known as Sp7) is another critical transcription factor expressed in osteoblast progenitors and osteoblasts. Reduced trabecular and cortical bone mass has been reported in Piezo1 SP7-Cre mice, which is markedly lower than Piezo1 Prx1-Cre mice (Zhou et al., 2020). Collagen type 1 (Col1) is an important structural protein of the ECM in bone and is expressed throughout the differentiation stages from preosteoblasts to mature osteoblasts. Piezo1 Col1-CreERT mice is characterized by increased bone resorption and decreased collagen expression, which leading to decreased bone density and changes in trabecular bone structure (Wang et al., 2020). Osteocalcin (OCN) is highly expressed in mature osteoblasts and plays a role in the regulation of bone mineralization and calcium ion homeostasis. Incomplete closure of cranial sutures was observed in Piezo1 OCN-Cre mice, accompanied with shorter weight-bearing long bones and significant bone mass loss (Sun et al., 2019).

Some studies have shown that Piezo1 regulates osteoblast differentiation under different forces, including HP loading (Sugimoto et al., 2017), static magnetic force (Hao et al., 2019), low-intensity pulsed ultrasound (LIPUS) (Zhang et al., 2021) and FSS (Song et al., 2020). Sun et al. (2019) found that mechanical-loading treatment can increase Piezo1 levels and osteoblasts function, while hind-limb suspension or simulated microgravity treatment can inhibit Piezo1 expression, impairing bone integrity and strength in mice. These results demonstrate that Piezo1 effect is correlated with mechanical force in osteoblasts.

### 3.3 Piezo1 in osteocytes

Osteocytes, mainly found in osteoblasts, are the predominant cellular inhabitants in bone tissue. The expression of Piezo1 is significantly higher in osteocytes than Piezo2 (Li et al., 2019). Fluid-flow stimulation on mature osteocytes can activate and upregulate Piezo1 channels (Li et al., 2019; Liu Z. et al., 2022). Yoda1 can enhance intracellular calcium mobilization and inhibit sclerostin (Sost) expression in osteocytes in a dose-dependent manner, thus promoting osteoblast differentiation (Sasaki et al., 2020). Whereas Piezo1 inactivation increases the expression of receptor activator of NF-κB ligand (RANKL) and osteoclasts number and decreases the expression of osteoprotegerin(OPG), thus promoting osteoclastogenesis (Li et al., 2019; Li et al., 2023). Furthermore, Piezo1 knockdown in osteocytes can decrease the bone formation-related genes (Alkaline Phosphatase, ALP) of osteoblasts induced by ultrasound stimulation in 3D osteocyte-osteoblast co-culture (Inoue et al., 2023).

Dentin matrix protein 1 (Dmp1) is a non-collagenous protein known to be an indicator of osteocytes. Compared to Piezo1 Runx2-Cre mice, Piezo1 Dmp1-Cre mice displayed a moderate reduction in trabecular and cortical bone mass. No significant spontaneous bone fractures were recorded (Li et al., 2019; Wang et al., 2020; Hendrickx et al., 2021). Similarly, Nottmeier et al. (2023) reported that Piezo1 deletion in osteocytes and osteoblasts suppresses the bone volume of the mandible and maxilla, as well as the height of the vertical alveolar bone. However, the morphology and length of the mandible and skull are unaffected by Piezo1 deletion.

### 3.4 Piezo1 in osteoclasts

Multinucleated osteoclasts are mainly found in myeloid hematopoietic precursors in the bone marrow (Boyle et al., 2003; de Vries et al., 2009). The mice with targeted deletion of Piezo1 in osteoclast lineage cells (Lyz2-Cre) (Hendrickx et al., 2021) and Ctsk-Cre (Wang et al., 2020) exhibited normal bone mass. Wang et al. (2020) also found that osteoblastic Piezo1 deficiency significantly decreases the levels of matrix proteins Col2α1 (alpha-1 type II collagen) and Col9α2 (alpha-2 type IX collagen) but markedly increases the number and activity of osteoclasts in the co-culture system of osteoclasts and Piezo-deficient osteoblasts. These findings suggest that Piezo1 can regulate osteoclast activation via type II and IX collagens, thus indirectly affecting bone resorption.

### 3.5 Piezo1 in PDLSCs and PDLFs

PDLSCs were initially isolated from human-impacted third molars (Seo et al., 2004). PDLSCs exhibit strong proliferation capability and high multilineage differentiation potential (Wang et al., 2011; Zhu and Liang, 2015). Mechanical force regulates the differentiation ability of PDLSCs and PDLFs and modifies associated genes (Panchamanon et al., 2019; Jin et al., 2020).

The Leptin receptor (Lepr) serves as a distinguishing factor for a distinct multipotent population of PDLSCs. Deletion of Piezo1 in Lepr + cells leads to a decrease in cellular cementum formation and alveolar bone mass, a lower ECM mass of cementum, and disorganized collagen fibrils. In contrast, femur bone mineral density are not affected. Hence, Piezo1 plays a crucial role in maintaining the equilibrium of the periodontium (Zhang et al., 2023). These findings underscore the role of Piezo1 in maintaining periodontal homeostasis.

Shen et al. (2020) showed that tension force activates and upregulates Piezo1. Besides, Piezo1 participates in periodontal ligament cells (PDLCs) mechanotransduction via the ERK signaling pathway. Piezo1 can also regulate osteoclastogenesis when the compression force is applied to PDLFs (Schröder et al., 2023). The release of adenosine triphosphate (ATP) and the activation of inflammatory genes during this process is also regulated by Piezo1 (Horie et al., 2023; Schröder et al., 2023). GsMTx4 treatment can significantly inhibit NF-kB activation and osteoclast-related factors induced by compression force, indicating that Piezo1 can regulate osteoclast differentiation via the NF-κB signaling pathway (Jin et al., 2015). Shen et al. (2023) also showed that Piezo1 inhibition can mitigate PDLFs apoptosis and damage under compression force by modulating the p38/ERK1/2 signaling pathway. In summary, Piezo1 participates in many processes that maintain the health and function of the periodontal ligament, including ATP release, osteoclastogenesis and osteogenesis.

## 4 Piezo1 signaling in bone remodeling

**FIGURE 4:**
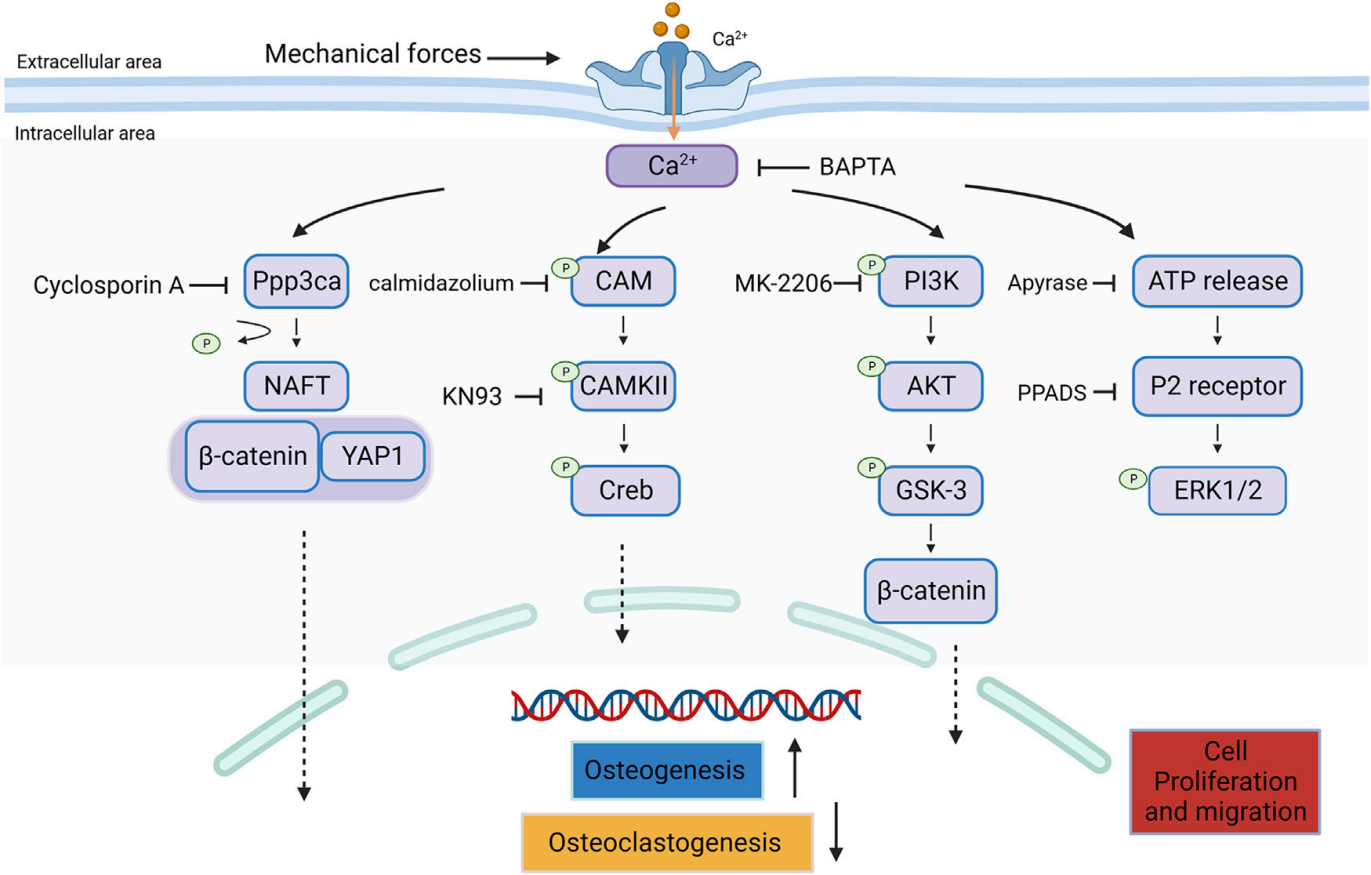
Signaling of Piezo1 in bone tissue.

Piezo1 mediates MA cationic currents and induces Ca^2+^ influx (Sun et al., 2019). This influx initiates downstream Ca^2+^ signaling, including the activation of the nuclear factor of activated T-cells (NFAT) (Zhou et al., 2020) and Ca^2+^-calmodulin (CaM)-dependent protein kinase (CaMKII) (Chen et al., 2022). The Piezo-Ca^2+^ signaling cascade plays a crucial in bone growth and significantly enhances the understanding of fundamental molecular and biological functions in the skeletal system. The signaling pathway of Piezo1 in bone tissue is summarized in Figure 4.

### 4.1 Piezo1 and NFAT

Studies have shown that the Ca^2+^/CaN/NFAT signaling pathway regulates bone formation and bone resorption (Tomita et al., 2002; Koga et al., 2005). Elevated Ca^2+^ concentrations activate calcineurin (CaN), leading to NFAT dephosphorylation and subsequent nuclear translocation (Ren et al., 2021).

CaN/NFAT1 signaling axis participated in Piezo1-mediated chondrocyte apoptosis, cartilage matrix production (Ren et al., 2023), fibrochondrogenesis (Yan et al., 2023) and vascular niche regeneration (Zhang et al., 2022).

Ppp3ca, also known as CaN, is a calcium and CaM -dependent serine/threonine protein phosphatase. Notably, Zhou et al. (2020) demonstrated that Piezo1 activation leads to NFAT, YAP, and β-catenin activation in BMSCs, and then regulates gene expression during osteoblast differentiation and bone formation, which can all be prevented by knocking down Piezo1. This implies a functional relationship between these proteins in bone formation and homeostasis.

### 4.2 Piezo1 and CAMKII

When intracellular calcium levels rise, calcium binds to CaM, which in turn binds to CaMKII, inducing its activation. This activation leads to autophosphorylation of CaMKII, and alters its conformation, allowing it to translocate and bind to different proteins within the cell (Rostas and Skelding, 2023). The phosphorylated CaMKII can then phosphorylate CREB, which is a transcription factor that regulates the expression of genes involved in numerous cellular processes (Yan et al., 2016).

Piezo1 modulates different biological processes through CAMKII, including blood pressure regulation (Zheng et al., 2022), immune response (Geng et al., 2021), perfusion recovery after ischemia (Xie et al., 2023), chronic inflammation (Liu H. L. et al., 2022) axon regeneration (Song et al., 2019) and cardiomyocyte hypertrophy (Yu et al., 2022).

In addition, CaMKII signaling is essential for Piezo1-mediated new bone formation in ankylosing spondylitis (Chen et al., 2022). Piezo1 also regulates osteoblast differentiation via CAMKII. CaMKII and Creb phosphorylation are downregulated in osteoblasts derived from Piezo1 OCN-Cre mice with lower osteoblast activity (Sun et al., 2019).

### 4.3 Piezo1 and YAP

Yes-associated protein (YAP) and its paralogue transcriptional coactivator with PDZ-binding motif (TAZ) are two highly related transcriptional cofactors in Hippo signaling (Dupont et al., 2011). The activity of YAP/TAZ is regulated by a complex interplay of mechanical and biochemical signals, including the tensional state of the F-actin cytoskeleton, cell-cell and cell-ECM adhesions, and interactions with other signaling pathways (Totaro et al., 2018). This YAP/TAZ axis can promote osteoblast differentiation by activating the downstream target Runx2 (Tang et al., 2016).

The increase in intracellular calcium levels can lead to the dephosphorylation and nuclear translocation of YAP, transforming it into a transcriptional co-activator (Sayedyahossein et al., 2023). Once in the nucleus, YAP collaborates with β-catenin, forming a YAP/β-catenin complex that directly interacts and upregulates osteogenic, chondrogenic, and angiogenic factors crucial for bone repair and regeneration (Liu Y. et al., 2022). In osteoblastic cells, Piezo1 controls the YAP-dependent expression of type II and IX collagens in response to mechanical loads, influencing osteoclast development in bone remodeling (Wang et al., 2020). In addition, YAP has been found to be decreased in degenerated cartilage (Sun et al., 2021b). The activation of Piezo1 via the YAP facilitates mechanically induced cartilage degradation (Feng et al., 2023).

However, some studies demonstrates that YAP could regulate Piezo1 expression in turn. Kong et al. (2022) found the nuclear localization of YAP could activated Piezo1 and enhance osteogenesis. Additionally, YAP, in collaboration with the G protein-coupled estrogen receptor (GPER) pathway, suppresses Piezo1 activation, thereby mitigating chondrocyte apoptosis (Sun et al., 2021b).

### 4.4 Piezo1 and β-catenin

Zhou et al. (2022) identified several pathways, including the Wnt/β-catenin and PI3K-Akt pathways to be potential targets of Piezo1 through bioinformatic analysis.

Wnt/β-catenin pathway promotes osteoblast development and proliferation (Gong et al., 2001; Hu et al., 2005). It is known to interact with the Hippo signaling pathway, specifically with the transcription factors YAP/TAZ, which are key components of the β-catenin degradation complex in the canonical Wnt signaling pathway. Upon Wnt/Frizzled binding, the destruction complex releases β-catenin and YAP/TAZ into the cytoplasm, triggering their subsequent translocation into the nucleus (Imajo et al., 2015; Totaro et al., 2018).

YAP and Piezo1 could serve as the downstream factor of Wnt5a, which work together to encourage the 3D cell intercalations that form the mandibular arch in mice (Tao et al., 2019). Another study suggested that Piezo1 promotes Wnt1 expression partly by activating YAP1 and TAZ, and then regulates bone development and homeostasis (Li et al., 2019).

Moreover, Piezo1 regulates the stemness of BMSCs through β-catenin. Blocking Wnt/β-catenin pathway via IWR-1 treatment inhibited the Yoda1-induced osteogenic differentiation of BMSCs (Hu et al., 2023). The involvement of Piezo1 in the proliferation and osteogenic differentiation of human dental follicle cells has been shown to be mediated by the Wnt/β-catenin signaling pathway (Xing et al., 2022).

### 4.5 Piezo1 and AKT

AKT plays a crucial role in cell survival, proliferation, growth, and metabolism (Manning and Toker, 2017). Notably, Sasaki et al. (2020) indicated that Piezo1 activation leads to Akt phosphorylation, subsequently down-regulating Sost. This process promotes bone formation, suggesting that Piezo1 participates in osteocyte mechanotransduction by triggering downstream Piezo1-Akt signaling. The canonical Wnt/β-catenin pathway involves the Ser and Thr protein kinase glycogen synthase kinase 3 (GSK3), initially recognized as an AKT substrate (Cross et al., 1995). Phosphor-GSK-3 prevents β-catenin degradation and facilitates nuclear translocation of accumulated β-catenin, regulating downstream target genes that control bone homeostasis, including Runx2 (Cai et al., 2016). FSS induces the expression of Runx-2 in MC3T3-E1 cells via the upregulation of Piezo1. This process involves the activation of the AKT/GSK-3β/β-catenin pathway to regulate bone formation under mechanical strain (Song et al., 2020).

Phosphorylated PI3K-AKT is related to Piezo1-mediated osteoblast maturation and ossification (Chen P. et al., 2021). Moreover, Piezo1-induced increase in intracellular calcium influx activates connexin 43 hemichannels (Cx43 HCs) in osteocytes through PI3K-Akt signaling under mechanical stress, thus regulating bone anabolic function (Zeng et al., 2022). Artemisinin (ART), a highly efficacious antimalarial drug, was found to exert therapeutic effects on osteoarthritis (OA) by acting on Piezo1 and AKT proteins. ART can downregulate Yoda1-induced upregulation of OA-related genes, and inhibit PI3K and AKT phosphorylation in chondrocytes (Gan et al., 2023).

### 4.6 Piezo1 and MAPK

Studies have reported that the MAPK signaling pathway participates in the regulation of osteogenic differentiation (Lu and Malemud, 2019). The C-terminal region of Piezo1, containing the domain that interacts with R-Ras, modulates the influx of calcium and activation of the ERK1/2 signaling pathway. This process regulates the osteoblastic development in BMSCs (Sugimoto et al., 2023). Piezo1 can also inhibit apoptosis of the human chondrocyte via the classic MAPK/ERK1/2 signal pathway (Li et al., 2016). Activation of the Piezo1 can induce ATP release and its binding with P2 receptor, and then enhances MSC migration. Researches have shown that process could be blocked by U0126, an inhibitor of the MEK/ERK signaling pathway, suggesting MEK/ERK signaling participates in Piezo1-mediated MSC migration (Mousawi et al., 2020).

It has been demonstrated that activation of Piezo channels in response to ultrasound stimulation can activate the MAPK pathway, particularly ERK1/2, in dental pulp stem cells (Gao et al., 2017). Shen et al. (2020) showed that blocking the Piezo1 channel can significantly increase the phosphorylation of GSK3α/β in PDLCs, indicating that GSK may be involved in PDLCs mechanotransduction. Also, GSK3 is a negative regulator of ERK1/2, c-Fos, and c-Jun (Wang et al., 2006; Götschel et al., 2008) and promotes β-catenin activation via Ras suppression (Liu et al., 2008). Nonetheless, further investigations should explore the potential interplay between the MAPK pathway and the Piezo1 channel in the above process.

## 5 Piezo1 and clinical therapy

### 5.1 Piezo1 and OP

OP is characterized by compromised bone strength, which substantially elevates the susceptibility to fractures, especially in the hip, spine, and wrist regions. OP is usually diagnosed after the fracture occurrence, and its etiology encompasses various elements, such as hormone fluctuations, the aging process, genetic predisposition, lifestyle choices, and certain medical disorders (Sheik Ali, 2023).

Polymorphisms in the Piezo gene are associated with human bone mineral density (BMD), a critical biomarker for the diagnosis and treatment of OP. A cross-phenotype meta-analysis for human BMD at various skeletal sites yielded the top 14 SNPs for Piezo1. Notably, the SNP rs62048221 was substantially correlated with BMD, especially around the heel, where mechanical force is applied during physical activities, such as standing. The T allele of this SNP was linked to BMD reduction, indicating that it can modulate the activity of cis-regulatory elements, thus influencing Piezo1 expression levels, which in turn affects BMD (Bai et al., 2020). OP patients have considerably lower levels of Piezo1 mRNA and protein than normal patients (Sun et al., 2019). Zhou et al. (2020) discovered that age is negatively correlated with gene expression of Piezo1 and Piezo 2 from human bone MSCs. Dzamukova et al. (2022) also discovered that mechanical forces, which increase with body weight during late adolescence, can trigger the Piezo1 activation, subsequently upregulating the kinase FAM20C within osteoblasts. Notably, FAM20C significantly regulates skeletal growth and bone mineralization by phosphorylating DMP1. Furthermore, simulated microgravity can decrease osteoblast function by inhibiting Piezo1 expression (Sun et al., 2019). Hu et al. (2023) revealed that the mitigation of bone loss in simulated microgravity conditions can be achieved through Piezo1 activation with Yoda1. Furthermore, Piezo1 activation induces a slight protective effect against bone loss in mice subjected to ovariectomy (OVX) and aging (Hu et al., 2023). Piezoelectric microvibration stimulation (PMVS) can alleviate OP induced by estrogen deficiency through Piezo1, MicroRNA-29a, and Wnt3a signaling pathways in osteoblasts, thus enhancing osteogenic activity and suppressing osteoclastic bone resorption (Wu et al., 2021). Furthermore, Sciancalepore et al. (2022) were the first to indicate that Piezo1 participates in the release of myokines. They also proposed the use of Yoda1 as a novel therapeutic intervention to augment the physiological advantages associated with exercise-induced myokine release. Guan et al. (2023) constructed a nanocarrier (ZOL-PLGA@Yoda1/SPIO) which combines the bone-targeting ability of Zoledronate (ZOL) and the magnetic properties of Superparamagnetic iron oxide (SPIO) to achieve dual-targeted administration and precise Piezo1-activated therapy for osteoporotic bone defects. *In vivo* and *in vitro* experiments have revealed that this nanocarrier not only enhances bone formation but also promotes the osteogenesis-angiogenesis coupling via the YAP/β-catenin signaling axis, providing a potentially effective strategy for the clinical treatment of osteoporotic bone defects.

The maintenance of alveolar bone homeostasis relies on occlusal force. The absence or reduction of occlusal force can lead to a disorder called alveolar bone disuse osteoporosis (ABDO), characterized by a net loss of alveolar bone. Furthermore, recombinant Slit guidance ligand 3 (SLIT3) protein into the periodontal ligament can stimulate Type H angiogenesis and osteogenesis by activating the Piezo1/Ca^2+^/HIF-1α/SLIT3 signaling pathway (Chen et al., 2023).

Moreover, Piezo1 is a novel biophysical intervention for OP caused by various factors, such as aging, diminished mechanical stimulation (microgravity), and estrogen insufficiency. Therefore, Piezo1 may be crucial for astronauts or persons who undergo protracted immobility for fractured bones.

### 5.2 Piezo1 and bone fracture

A bone fracture is widely caused by significant mechanical force or strain, such as falling, vehicular collisions, or athletic traumas. Nevertheless, specific medical diseases, such as osteoporosis and certain cancer types, can compromise bone strength, rendering them more vulnerable to fractures, even when subjected to modest pressure. Notably, the duration of the healing process often spans from 4 to 8 weeks depending on age, overall health, and the specific nature of the fracture (Einhorn and Gerstenfeld, 2015).

Piezo1 downregulation impairs fracture healing in the callus (Chen P. et al., 2021), while Piezo1 chemical activation by Yoda1 enhances fracture healing by stimulating periosteal stem cells (PSCs)-modulated chondrogenesis and osteogenesis and expediting the transformation of cartilage into bone (Liu Y. et al., 2022). Moreover, Piezo1 activation can increase the expression of vascular endothelial growth factor A, suggesting that Piezo1 may have a secondary function in angiogenesis, which creates new blood vessels to feed oxygen and nutrients to the fracture site (Liu Y. et al., 2022).

Higher-intensity ultrasound can effectively accelerate fracture healing, particularly in a mouse osteoporotic fracture model, by accelerating the process of endochondral ossification through Piezo1 activation. However, Piezo1 inhibition by a specific inhibitor (GsMTx4) negatively affects fracture healing induced by ultrasound exposure (Inoue et al., 2023). Besides, Piezo1 can sense LIPUS and regulate the proliferation of osteoblasts by triggering the activation of ERK1/2 phosphorylation and perinuclear F-actin polymerization, indicating that Piezo1 can enhance fracture repair (Zhang et al., 2021).

### 5.3 Piezo1 and cancer

Breast cancer metastasis, particularly in the bone, significantly limits cancer treatment. Piezo1 regulates cancer cell migration and invasion by modulating cell adhesion, stiffness, and contractility, thus influencing invadopodia formation and MMP expression (Karska et al., 2023). Piezo1 modulates breast cancer metastases in the bone by affecting osteoclast and osteocyte activity. The Piezo1 ion channel is essential for osteocyte mechanotransduction. Besides, the chemical activation of Piezo1 ion channel enhances the capacity of osteocyte to prevent cancer extravasation under low-magnitude high-frequency (LMHF) vibration (Song et al., 2022). Cancer cells can penetrate and spread to bone through osteoclasts, which are cells that tear down bone tissue. Piezo1 stimulation improves osteocytes’ mechanoresponse to LMHF vibration, thus inhibiting osteoclastogenesis and decreasing MDA-MB-231, a type of breast cancer cell, from migrating (Lin C. Y. et al., 2022). In addition, Piezo1 is highly expressed in osteosarcoma (OS) cells and regulated apoptosis, invasion, and cell proliferation of OS cells (Jiang et al., 2017).

### 5.4 Piezo1 and tooth movement

Understanding the mechanism of alveolar bone remodeling under mechanical force is a primary concern in orthodontics. Alveolar bone and periodontal ligament (PDL) are closely related structures in periodontium development and mechanotransduction during orthodontic tooth movement (OTM). PDL, a vital connective and supporting tissue, attaches the tooth to the adjacent bone through collagen fiber bundles, enabling the tooth to disperse and withstand loading force, including the masticatory and orthodontic force. Osteoclasts, osteoblasts, osteocytes, periodontal ligament fibroblasts, and periodontal ligament stem cells in the periodontium function as sensory cells and effectors, converting mechanical force into intracellular signals and facilitating tooth movement induced by orthodontic force (Jiang et al., 2016; Zhou et al., 2022).

**FIGURE 5:**
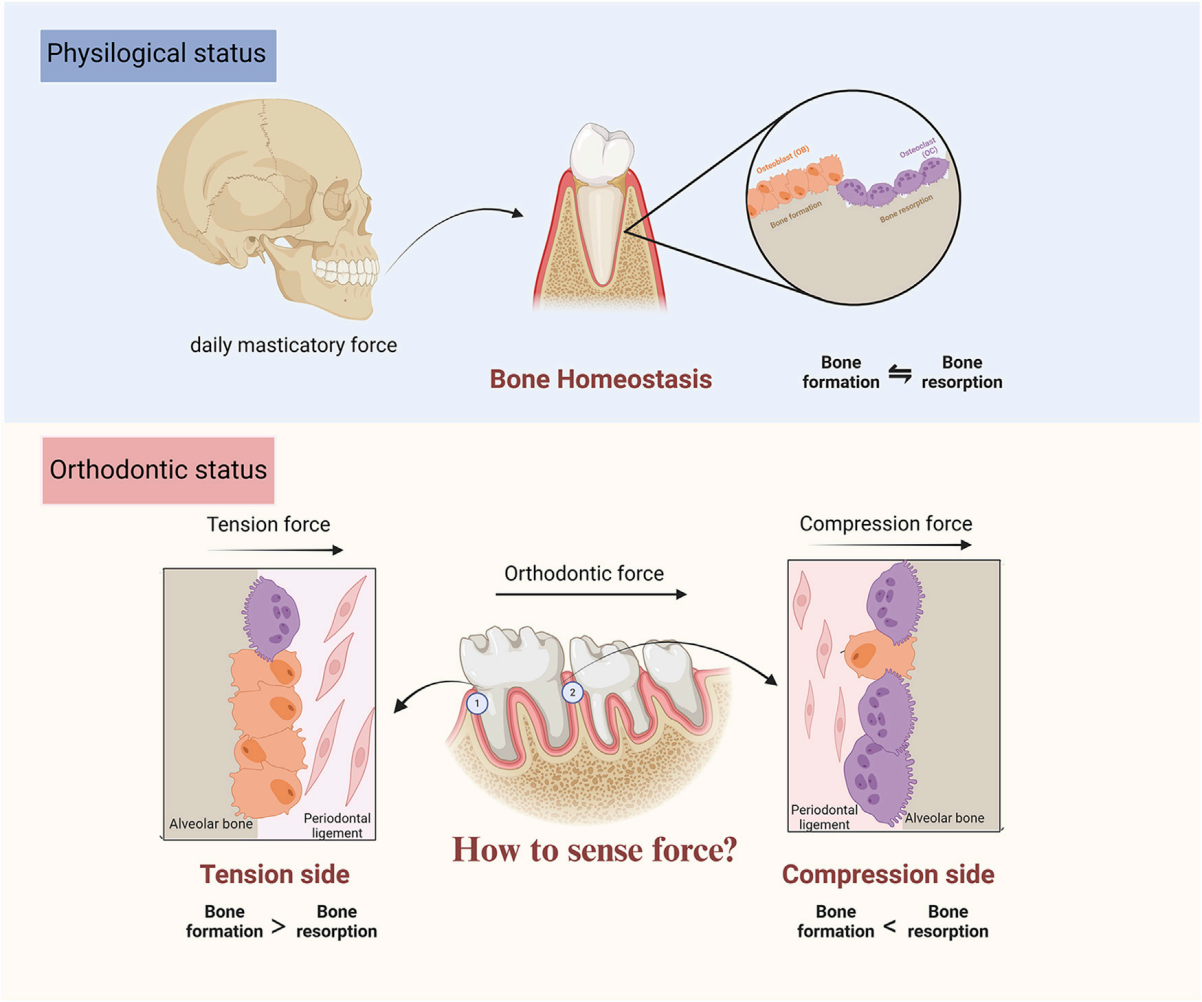
Illustration of orthodontic tooth movement process.

The established pressure-tension hypothesis indicates that orthodontic force induces PDL compression in certain areas where blood flow is reduced, and PDL stretch in others where the blood flow is enhanced or maintained. Different force stimuli in the PDL result in diverse biological reactions and chemical environments, including oxygen concentration and transcription factor levels, leading to bone resorption on the compression side and bone creation on the tension side (Meeran, 2012; McCormack et al., 2014) (Figure 5).

Piezo1 exhibits intense immunoreactivity in both human and murine periodontal ligaments (Kang et al., 2014; Gao et al., 2017; Gaite et al., 2023). Besides, Piezo1 is crucial for sustaining the rate of OTM and promoting alveolar bone remodeling on the tension side (Jiang Y. K. et al., 2021; Du and Yang, 2023). However, Nottmeier et al. (2023) proposed a different opinion that Piezo1 impairment has minimal effect on the tooth movement distance by establishing an OTM rat model. Nonetheless, a longer testing period may reveal significant variation in bone remodeling since the OTM model was only tested for 12 days.

Furthermore, Piezo1 offers a strong theoretical foundation for the potential use of 3D-printed implants in orthopedic surgery. The low stiffness of the three-dimensionally printed Ti2448 promoted angiogenesis and osteogenesis by enhancing the Piezo1/YAP signaling axis, which in turn regulated macrophage polarization (Tang et al., 2021).

## 6 Discussion and conclusion

**FIGURE 6:**
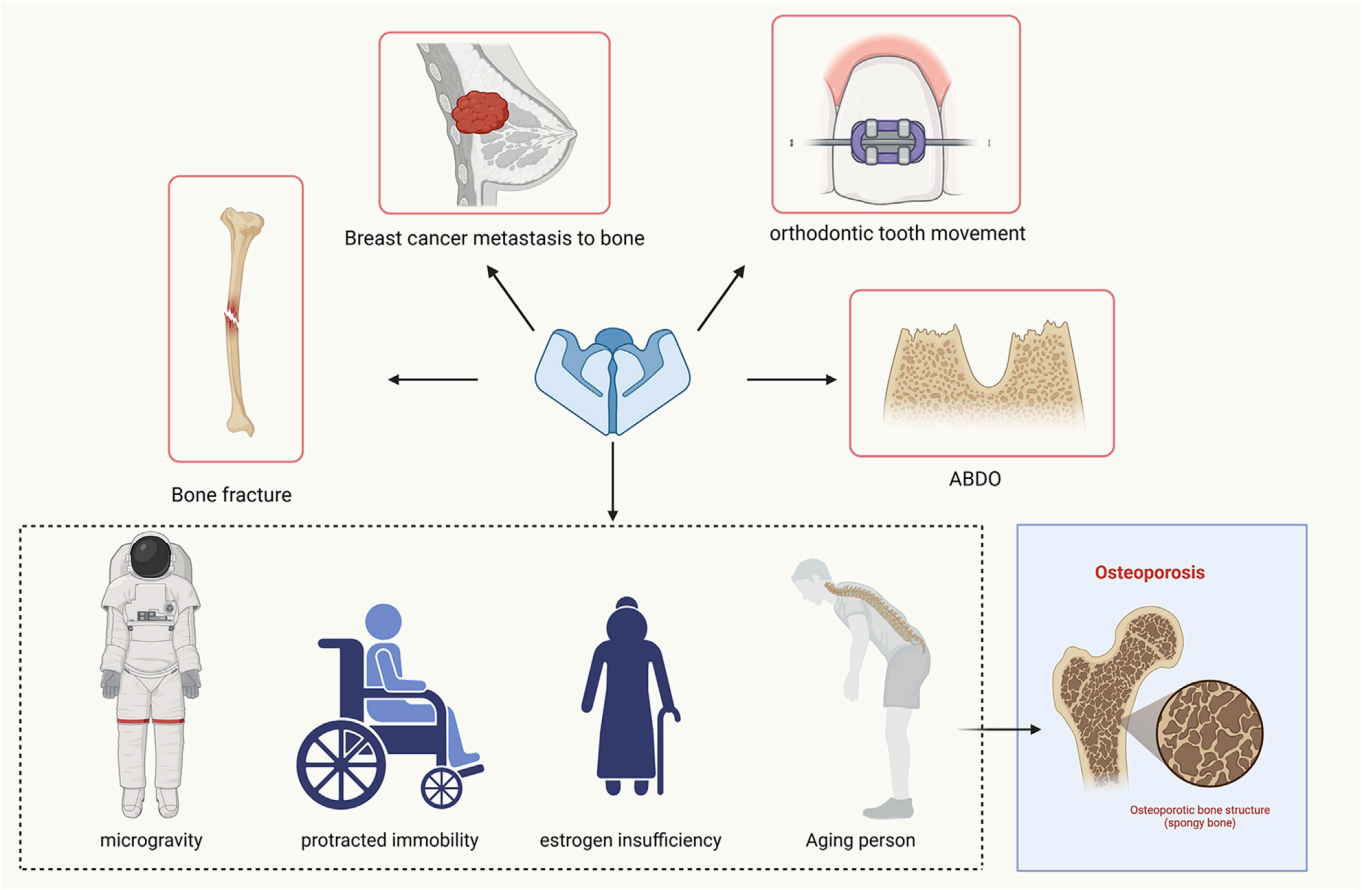
Piezo1 and related bone diseases.

Recent research has made notable advancements in uncovering the distinct structure and function of Piezo1 in various tissues and animals. Emerging evidence suggests that Piezo1 can detect mechanical stress and convert it into biological signals, thereby maintaining bone homeostasis. The latest research on the function of the Piezo1 channel in bone remodeling is thoroughly reviewed in this article. However, further investigation is essential to fully comprehend the underlying processes behind Piezo1-mediated bone remodeling. Such insights hold promising solutions for bone diseases and may expedite advancements in OTM techniques (Figure 6).
